# Replication and Characterization of Association between *ABO* SNPs and Red Blood Cell Traits by Meta-Analysis in Europeans

**DOI:** 10.1371/journal.pone.0156914

**Published:** 2016-06-09

**Authors:** Stela McLachlan, Claudia Giambartolomei, Jon White, Pimphen Charoen, Andrew Wong, Chris Finan, Jorgen Engmann, Tina Shah, Micha Hersch, Clara Podmore, Alana Cavadino, Barbara J. Jefferis, Caroline E. Dale, Elina Hypponen, Richard W. Morris, Juan P. Casas, Meena Kumari, Yoav Ben-Shlomo, Tom R. Gaunt, Fotios Drenos, Claudia Langenberg, Diana Kuh, Mika Kivimaki, Rico Rueedi, Gerard Waeber, Aroon D. Hingorani, Jacqueline F. Price, Ann P. Walker, UCLEB Consortium

**Affiliations:** 1 Centre for Population Health Sciences, Usher Institute of Population Health Sciences and Informatics, University of Edinburgh, Edinburgh, United Kingdom; 2 Department of Psychiatry, Icahn Institute for Genomics and Multiscale Biology, Icahn School of Medicine at Mount Sinai, The Leon and Norma Hess Center for Science and Medicine, New York, New York, United States of America; 3 University College London Genetics Institute, Department of Genetics, Environment and Evolution, London, United Kingdom; 4 Department of Non-communicable Disease Epidemiology, Faculty of Epidemiology and Population Health, London School of Hygiene and Tropical Medicine, London, United Kingdom; 5 Department of Tropical Hygiene, Faculty of Tropical Medicine, Mahidol University, Bangkok, Thailand; 6 MRC Unit for Lifelong Health and Ageing at UCL, London, United Kingdom; 7 Institute of Cardiovascular Science, University College London, London, United Kingdom; 8 Genetic Epidemiology Group, Institute of Cardiovascular Science, University College London, London, United Kingdom; 9 Farr Institute of Health Informatics Research, Department of Epidemiology & Public Health, University College London, London, United Kingdom; 10 Department of Medical Genetics, University of Lausanne, Lausanne, Switzerland; 11 Swiss Institute of Bioinformatics, Lausanne, Switzerland; 12 MRC Epidemiology Unit, University of Cambridge School of Clinical Medicine, Institute of Metabolic Science, Cambridge Biomedical Campus, Cambridge, United Kingdom; 13 Centre for Environmental and Preventive Medicine, Wolfson Institute of Preventive Medicine, Queen Mary University of London, London, United Kingdom; 14 Population, Policy and Practice, UCL Institute of Child Health, University College London, London, United Kingdom; 15 Department of Primary Care & Population Health, UCL Institute of Epidemiology & Health Care, University College London, London, United Kingdom; 16 Centre for Population Health Research, School of Health Sciences and Sansom Institute of Health Research, University of South Australia, Adelaide, Australia; 17 South Australian Health and Medical Research Institute, Adelaide, Australia; 18 School of Social and Community Medicine, University of Bristol, Bristol, United Kingdom; 19 Institute of Health Informatics, University College London, London, United Kingdom; 20 Institute for Social and Economic Research, University of Essex, Colchester, United Kingdom; 21 Department of Epidemiology & Public Health, UCL Institute of Epidemiology & Health Care, University College London, London, United Kingdom; 22 MRC Integrative Epidemiology Unit, School of Social and Community Medicine, University of Bristol, Bristol, United Kingdom; 23 Centre for Cardiovascular Genetics, Institute of Cardiovascular Science, University College London, London, United Kingdom; 24 Department of Internal Medicine, Centre Hospitalier Universitaire Vaudois and University of Lausanne, Lausanne, Switzerland; Roswell Park Cancer Institute, UNITED STATES

## Abstract

Red blood cell (RBC) traits are routinely measured in clinical practice as important markers of health. Deviations from the physiological ranges are usually a sign of disease, although variation between healthy individuals also occurs, at least partly due to genetic factors. Recent large scale genetic studies identified loci associated with one or more of these traits; further characterization of known loci and identification of new loci is necessary to better understand their role in health and disease and to identify potential molecular mechanisms. We performed meta-analysis of Metabochip association results for six RBC traits—hemoglobin concentration (Hb), hematocrit (Hct), mean corpuscular hemoglobin (MCH), mean corpuscular hemoglobin concentration (MCHC), mean corpuscular volume (MCV) and red blood cell count (RCC)—in 11 093 Europeans from seven studies of the UCL-LSHTM-Edinburgh-Bristol (UCLEB) Consortium. We identified 394 non-overlapping SNPs in five loci at genome-wide significance: 6p22.1-6p21.33 (with *HFE* among others), 6q23.2 (with *HBS1L* among others), 6q23.3 (contains no genes), 9q34.3 (only *ABO* gene) and 22q13.1 (with *TMPRSS6* among others), replicating previous findings of association with RBC traits at these loci and extending them by imputation to 1000 Genomes. We further characterized associations between *ABO* SNPs and three traits: hemoglobin, hematocrit and red blood cell count, replicating them in an independent cohort. Conditional analyses indicated the independent association of each of these traits with *ABO* SNPs and a role for blood group O in mediating the association. The 15 most significant RBC-associated *ABO* SNPs were also associated with five cardiometabolic traits, with discordance in the direction of effect between groups of traits, suggesting that *ABO* may act through more than one mechanism to influence cardiometabolic risk.

## Introduction

Red blood cell (RBC) traits are routinely measured in clinical practice. These traits, hemoglobin concentration (Hb), hematocrit (Hct), mean corpuscular hemoglobin (MCH), mean corpuscular hemoglobin concentration (MCHC), mean corpuscular volume (MCV) and red blood cell count (RCC) are tightly regulated so that deviations from the physiological ranges are usually a sign of disease, such as hematological, infectious and immune disorders and cancer. Therefore, they serve as markers not only of specific hematological conditions but also of the general health of an individual. However, physiological values for the traits also vary between healthy individuals and between different ethnic groups, at least in part due to genetic factors [1–13].

Heritability varies between the different RBC traits, with estimates ranging from 40–80% for Hb and RCC, although values as low as 14% for MCH and as high as 96% for MCV have been reported [14–17]. Only a few large scale genetic association studies of RBC traits have been performed to date, the latest one identifying 75 loci potentially influencing one or more of these traits [13]. The largest studies were of Caucasian/European cohorts, followed by studies of African Americans and Asians, with several of the RBC-associated loci consistently identified across different ethnic groups. The recent use of large scale meta-analyses has accelerated the detection of loci and has emphasized the need for additional studies to identify still undetected contributors to the underlying genetic basis of these traits [1–13]; reviewed in [18, 19]. Further characterization of known loci is equally important in order to better understand their roles in erythropoiesis, susceptibility to and development of different disorders and their impact on the clinical characteristics and prognosis in those disorders [18–20].

In this study, RBC traits were associated with five loci, including 11 new SNPs for *ABO* (the gene encoding ABO blood group), further refining these associations. Conditional analyses point to the role of *ABO* SNPs predicting blood group O in the association with Hb, Hct and RCC and to the independent association of all three traits with the *ABO* locus. *ABO* is a highly pleiotropic locus; direct comparison of results for the 15 most significant RBC-associated *ABO* SNPs versus additional cardiometabolic traits showed that RBC (Hct, Hb, RCC) and liver (logALP) traits exhibited a negative direction of effect whereas some lipid (LDL, TC) and coagulation factor traits (logVWF, FVIII) had a positive effect, indicating the wide influence of the *ABO* locus upon cardiometabolic risk.

## Results

### Meta-analysis in the UCLEB consortium

The study characteristics are given in Table 1. The analysis was done on ~4.2 million genotyped and imputed SNPs. An identical analysis was performed for each study and trait, accounting for the covariates available for that specific study, before combining the results by meta-analysis. Genomic-control inflation factors (λ_GC_) varied between 0.987 for Hb in MRC NSHD to 1.041 for RCC in BWHHS in individual studies; these were used to correct the test statistics in the meta-analysis (S2 Table). Manhattan plots for the meta-analysis of each trait are shown in S2 Fig. The corresponding Q-Q plots are shown in S3 Fig.

**Table 1:** Study characteristics.

Study	N	F, %	Age, year	T2D, %	Ever smoked, %	eGFR, mL/min/1.73m^2^	Hb, g/dL	Hct, %	MCH, pg	MCHC, g/dL	MCV, fL	RCC, ×10^12^/L
**BRHS**	2310	0	68.89 (5.62)	12	73	63.13 (11.15)	14.56 (1.21)	45.09 (3.49)	30.13 (1.89)	32.33 (1.16)	93.23 (5.36)	4.84 (0.41)
**BWHHS**	1884	100	70.73 (5.31)	15	47	66.81 (11.53)	13.45 (1.07)	41.70 (3.20)	29.51 (1.79)	32.30 (1.19)^b^	91.40 (5.05)	4.57 (0.38)
**CaPS**	1268	0	56.78 (4.46)	12	80	69.32 (11.93)	14.94 (1.07)	44.65 (3.17)	30.88 (1.83)	33.50 (0.81) ^b^	92.16 (4.78)	4.85 (0.36)
**ELSA**	1881	47	73.69 (9.45)	14	67		14.25 (1.50)					
**ET2DS**	996	49	67.89 (4.24)	100	61	70.42 (19.03)	14.06 (1.49)	40.62 (4.18)	31.31 (1.96) ^b^	34.61 (0.67) ^b^	90.43 (4.87)	4.50 (0.48)
**MRC NSHD**	1672	52	63.29 (1.11)	8^a^	70 ^a^	92.96 (22.19)	14.21 (1.22)	41.87 (3.41)	30.63 (1.73)	34.11 (1.15) /33.95 (1.13) ^b^	90.26 (4.72)	4.65 (0.41)
**WHII**	1082	22	43.78 (5.87)				14.60 (1.16)					
**Total N**	11 093	-	-	-	-	-	11 093	8127	8125 ^b^	8125 ^b^	5817	8128

Data are mean (SD). The seven studies included in the analysis are: British Regional Heart Study (BRHS), British Women’s Heart and Health Study (BWHHS), Caerphilly Prospective Study (CaPS), English Longitudinal Study of Ageing (ELSA), Edinburgh Type 2 Diabetes Study (ET2DS), MRC National Survey of Health and Development (MRC NSHD) and the Whitehall II Study (WHII). Study characteristics are given for: number of participants with at least one red blood cell trait available (N), percentage of females in the study (F), participants’ mean age (Age), percentage of participants with confirmed type 2 diabetes (T2D), percentage of people who currently smoke or have previously smoked (Ever smoked), estimated glomerular filtration rate (eGFR), hemoglobin (Hb), hematocrit (Hct), mean corpuscular hemoglobin (MCH), mean corpuscular hemoglobin concentration (MCHC), mean corpuscular volume (MCV) and red blood cell count (RCC).

^a^ Data from a different data collection wave conducted between six to nine years before.

^b^ Calculated traits are shown in red; the MRC NSHD results shown as “b” include 419 participants for whom an observed MCHC value was not available, instead it was calculated.

The 736 statistically significant single SNP-trait associations are listed in S3 Table. In total, there were 394 non-overlapping SNPs in five loci which reached the genome-wide significance threshold of 5×10^−8^ used in this study (116 SNPs associated with Hb in three loci; 15 with Hct in one locus; 371 with MCH in three loci; six with MCHC in one locus; 188 with MCV in four loci; and 40 with RCC in two loci). 338 of these SNPs (86%) were significantly associated with more than one RBC trait. The five loci with SNPs reaching statistical significance were: 6p22.1-6p21.33 (locus includes *HFE* as a primary candidate gene), 6q23.2 (includes *HBS1L* gene among others), 6q23.3 (locus does not contain any genes), 9q34.3 (includes only the *ABO* gene) and 22q13.1 (includes the *TMPRSS6* gene among others). Overlap of the significant loci for all six RBC traits is shown in S4 Fig.

The 6p22.1-6p21.33 locus includes 181 genes over ~2.3 Mb and was significantly associated with Hb, MCH and MCV. The lead SNP (with the lowest *P* value) for all three traits was rs80215559 in *SLC17A2* gene (*P* = 8.90×10^−14^, *P* = 2.42×10^−21^ and *P* = 1.85×10^−15^, respectively). However, the *HFE* C282Y functional variant has an extended founder haplotype, therefore, *HFE* is considered to be the primary candidate gene in this locus, previously associated with all three RBC traits [3–9, 12, 13].

MCH, MCV and RCC were significantly associated with SNPs in the 6q23.2 locus, which is approximately 272 Kb in size and contains two genes, one pseudogene and a microRNA (*ALDH8A1*, *HBS1L*, *MEMO1P2* and *MIR3662*, respectively). Lead SNPs in this locus were rs34164109 for MCH (*P* = 4.52×10^−17^), rs35959442 for MCV (*P* = 2.44×10^−15^) and rs35786788 for RCC (*P* = 2.47×10^−19^). One of the genes in this locus (*HBS1L)* has been repeatedly reported to be associated with all three traits as part of the *HBS1L-MYB* region [5–9, 11–13].

The signal at 6q23.3 had only two SNPs reaching the significance threshold, (rs592423 and rs590856) and the lead association for both was with MCV (*P* = 1.90×10^−8^ and *P* = 2.45×10^−8^, respectively). Both SNPs were also associated with MCH and a further 23 SNPs were associated with MCV at the suggestive threshold of statistical significance. These findings confirm the previous association of this locus with MCH and MCV [6, 7, 9, 13]. There are no known genes in this locus, however the closely located *CITED2* has been reported as a potential candidate gene.

The 9q34.3 locus contains only the *ABO* gene and is approximately 16 Kb in size. SNPs in this locus were associated with Hb, Hct and RCC. rs507666 was the lead SNP for Hb (*P* = 1.84×10^−10^), rs8176643 for Hct (*P* = 9.64×10^−12^) and rs8176685 for RCC (*P* = 1.98×10^−8^).

SNPs in the 22q13.1 locus (~ 86 Kb in size) were associated with Hb, MCH, MCHC and MCV. This locus contains four genes, however, *TMPRSS6* is a primary candidate gene and has been previously identified as associated with all four RBC traits [2, 3, 5–9, 11–13, 21]. The lead SNPs associated with these four traits in the present study were all in *TMPRSS6*: rs877908 for Hb (*P* = 1.27×10^−8^), rs855791 for MCH and MCV (*P* = 1.57×10^−24^ and *P* = 5.40×10^−16^, respectively), and rs2413450 for MCHC (*P* = 2.15×10^−8^).

There was a strong positive correlation between hemoglobin, hematocrit and red blood cell count as well as between mean corpuscular hemoglobin and mean corpuscular volume (*r* > 0.75; S6 Table). All other RBC trait combinations show weak to moderate positive or negative correlation with each other (S6 Table).

### Replication analysis of *ABO* locus in the CoLaus study

For replication, 47 SNPs were selected in/around the *ABO* gene. All of these were below the suggestive threshold for Hb, Hct, and RCC in the meta-analysis, out of which 15 were below the significance threshold. The CoLaus study had 2848 participants for whom both genotypes and phenotypes were available. Using a nominal significance cut-off of *P* < 0.05 for replication, we confirmed the association between four significant SNPs and Hb, and between 10 significant SNPs and Hct (Table 2). Two significant associations of SNPs with RCC were not replicated. Likewise, associations between five SNPs (rs600038, rs651007, rs579459, rs649129 and rs495828) and Hb and Hct were not replicated. However, CoLaus was previously part of a larger effort to identify loci associated with RBC traits in 71 861 individuals of European or South Asian ancestry [13]. Four out of five SNPs were present and highly significant in their analysis for Hb, Hct and RCC, respectively: rs651007 *P* = 3.82×10^−14^, 1.26×10^−12^ and 1.61×10^−14^; rs579459 *P* = 1.35×10^−15^, 7.59 ×10^−14^ and 9.25×10^−18^; rs649129 *P* = 1.46 ×10^−15^, 1.54×10^−13^ and 9.77×10^−16^; rs495828 *P* = 1.59 ×10^−15^, 1.48×10^−13^ and 1.39×10^−15^, giving confidence that the association between these SNPs and traits is genuine. For Hct and RCC, 29 and two suggestive associations, respectively, were also replicated. We used the same nominal significance cut-off of *P* < 0.05 to call these associations replicated as they were in a locus already known to be associated with the traits (S4 Table).

**Table 2:** Results of meta-analysis and replication analysis for 15 most significant SNPs in the *ABO* locus.

					Meta-analysis (N_Hb_ = 11 093; N_Hct_ = 8128; N_RCC_ = 8129)			Replication (N = 2848)		
rs#	allele	chr	bp	trait	β	SE	*P*	β	SE	*P*
**rs8176685**	**d**	**9**	**136138765**	**Hb**	**-0.1328**	**0.0232**	**1.08×10–8**	**-0.1000**	**0.0543**	**0.06547**
				Hct	-0.5406	0.0802	1.59×10^−11^	-0.3278	0.1502	**0.02909**
				RCC	-0.0533	0.0095	1.98×10^−8^	-0.0307	0.0197	0.11930
**rs149092047**	d	9	136139907	Hb	-0.1008	0.0173	5.29×10^−9^	-0.0782	0.0365	**0.03198**
				Hct	-0.3519	0.0588	2.22×10^−9^	-0.2480	0.1009	**0.01396**
**rs2519093**	t	9	136141870	Hb	-0.1217	0.0193	2.67×10^−10^	-0.0754	0.0406	0.06335
				Hct	-0.4398	0.0655	1.83×10^−11^	-0.2561	0.1123	**0.02261**
**rs28850884**	d	9	136145424	Hb	-0.1035	0.0178	6.26×10^−9^	-0.0963	0.0418	**0.02129**
				Hct	-0.3576	0.061	4.52×10^−9^	-0.2965	0.1158	**0.01041**
**rs9411378**	a	9	136145425	Hb	-0.1033	0.0178	6.47×10^−9^	-0.0962	0.0417	**0.02120**
				Hct	-0.3576	0.0609	4.22×10^−9^	-0.2952	0.1155	**0.01058**
**rs550057**	t	9	136146597	Hb	-0.0982	0.0172	1.12×10^−8^	-0.0793	0.0361	**0.02795**
				Hct	-0.3454	0.0585	3.57×10^−9^	-0.2490	0.0998	**0.01257**
**rs507666**	a	9	136149399	Hb	-0.1228	0.0193	1.84×10^−10^	-0.0716	0.0399	0.07251
				Hct	-0.4441	0.0655	1.19×10^−11^	-0.2465	0.1104	**0.02554**
**rs8176643**	d	9	136149709	Hb	-0.1253	0.0199	3.37×10^−10^	-0.0802	0.0442	0.06930
				Hct	-0.4607	0.0676	9.64×10^−12^	-0.2682	0.1222	**0.02818**
				RCC	-0.0436	0.008	4.98×10^−8^	-0.0255	0.0160	0.11180
**rs532436**	a	9	136149830	Hb	-0.1219	0.0193	2.44×10^−10^	-0.0717	0.0399	0.07242
				Hct	-0.4418	0.0654	1.47×10^−11^	-0.2464	0.1104	**0.02557**
rs600038	c	9	136151806	Hb	-0.1021	0.0185	3.47×10^−8^	-0.0320	0.0381	0.40180
				Hct	-0.3797	0.0628	1.46×10^−9^	-0.1355	0.1055	0.19920
rs651007^a^	t	9	136153875	Hb	-0.1046	0.0185	1.62×10^−8^	-0.0329	0.0380	0.38610
				Hct	-0.3821	0.0628	1.15×10^−9^	-0.1393	0.1051	0.18500
rs579459^a^	c	9	136154168	Hb	-0.1045	0.0185	1.63×10^−8^	-0.0326	0.0382	0.39290
				Hct	-0.3820	0.0628	1.16×10^−9^	-0.1376	0.1056	0.19250
rs649129^a^	t	9	136154304	Hb	-0.1053	0.0185	1.29×10^−8^	-0.0324	0.0382	0.39550
				Hct	-0.3822	0.0628	1.14×10^−9^	-0.1372	0.1056	0.19390
rs495828^a^	t	9	136154867	Hb	-0.1051	0.0185	1.37×10^−8^	-0.0324	0.0382	0.39660
				Hct	-0.3821	0.0628	1.17×10^−9^	-0.1372	0.1056	0.19400
**rs635634**	t	9	136155000	Hb	-0.1205	0.0193	3.86×10^−10^	-0.0722	0.0399	0.07080
				Hct	-0.4393	0.0654	1.90×10^−11^	-0.2498	0.1105	**0.02375**

All SNPs were significantly associated (*P* < 5×10^−8^) with one or more red blood cell traits in seven studies from the UCLEB consortium. For every SNP (rs#) minor (risk) allele, chromosome (chr) and position (bp) on the human genome build 19 are given. Effect size (β), corresponding standard error (SE) and *P* value are shown for each trait associated with the SNP. For replication analysis, SNPs meeting nominal significance cut-off of *P* < 0.05 are marked in bold.

^**a**^ Four SNPs previously reported as associated with RBC traits.

### Conditional analyses in *HFE* locus

Conditional analyses were performed using the two major *HFE* mutations (C282Y and H63D) as covariates, to test if the signal in this locus was due to *HFE* alone. C282Y and H63D lie on different ancestral haplotypes, which in case of C282Y may extend over 6 Mb and to around 700 kb or less for H63D [22–24]. The strong peak in this region, containing many SNPs, could be due to the association of *HFE* mutations, particularly C282Y, with long founder haplotypes. For all three traits, the signal in this locus disappeared in conditional analysis, indicating that these two mutations of *HFE* are responsible for all of the observed significant association (S5 Fig). For Hb however, a suggestive signal (spanning ~480 Kb and containing 28 genes) was seen in the main analysis within the major histocompatibility complex locus at 6p21.32-6p21.31, centromeric to *HFE*. This locus remained at the suggestive significance threshold in the conditional analysis (lead SNP rs2734573, *P* = 1.33×10^−6^; S5 Fig).

### Conditional analyses in *ABO* locus

#### Conditioning on blood groups

**Fig 1:**
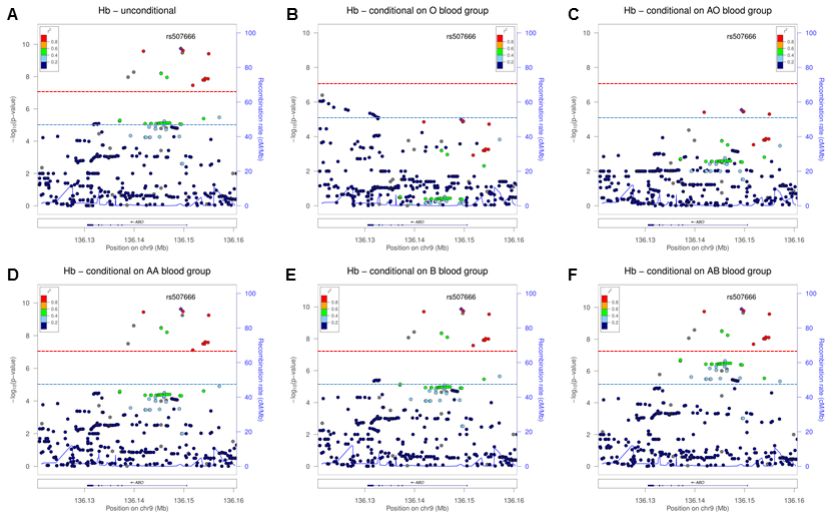
Meta-analysis association results for hemoglobin in unconditional and conditional analyses in the *ABO* locus. Regional plots show (A) unconditional analysis and analysis conditional on (B) O, (C) AO, (D) AA, (E) B and (F) AB blood group haplotype. The most significant SNP in the unconditional analysis, rs507666, is highlighted throughout to facilitate comparison of results. The color coding of the LD between the SNPs ranges from dark blue for r^2^ = 0–0.2 to red for r^2^ = 0.8–1, and is grey where LD information was not available. Blue line represents suggestive and red line significant threshold.

At the *ABO* locus, using Hb as an example, analysis conditional on O blood group haplotype caused the signal at ~135.154 Mb (with rs507666 as lead SNP) to drop under the suggestive threshold while some SNPs at ~136.131 Mb and proximal became significant at the suggestive level (Fig 1B) compared to the unconditional analysis. Similarly, conditioning on AO blood group haplotype caused the same signal at ~135.154 Mb to drop around and below the suggestive threshold (Fig 1C). Analyses conditional on AA and B blood group haplotypes did not differ from the unconditional analysis (Fig 1D and 1E), while in analysis conditional on AB blood group haplotype a group of SNPs directly proximal to rs507666 increased in significance (Fig 1F). However, just as in the unconditional analysis, only three SNPs from this group were above the significance threshold: rs28850884, rs9411378 and rs550057. Analyses run separately on BO and BB blood group haplotypes did not differ from those in unconditional analysis and so were combined. The distribution of the three traits associated with the *ABO* locus according to blood groups is shown in Table 3, with each trait showing highly significant (*p*≤ 0.0001) variation across the blood groups.

**Table 3:** One-way analysis of variance in hemoglobin (Hb), hematocrit (Hct) and red blood cell count (RCC) between different blood groups, as derived based on three SNP haplotypes.

**Blood group**	**N**_**Hb**_	**Hb**	**N**_**hct & RCC**_	**Hct**	**RCC**
**AA**	890	14.18 (1.26)	659	42.76 (3.77)	4.68 (0.43)
**AO**	4113	14.19 (1.35)	3007	42.80 (3.86)	4.67 (0.43)
**AB**	394	14.29 (1.33)	282	42.95 (3.95)	4.70 (0.46)
**O**	4704	14.33 (1.32)	3434	43.23 (3.86)	4.71 (0.42)
**B**	992	14.34 (1.34)	746	43.22 (3.88)	4.75 (0.44)
**P**		**4.97×10**^**−06**^		**4.99×10**^**−05**^	**0.0001**

Data are number of participants in each group (N) and mean (SD) for each trait. All differences between the groups were highly significant (p≤ 0.0001, in bold). Blood groups are ordered by the increasing value of Hb.

#### Conditioning on associated RBC traits

**Fig 2:**
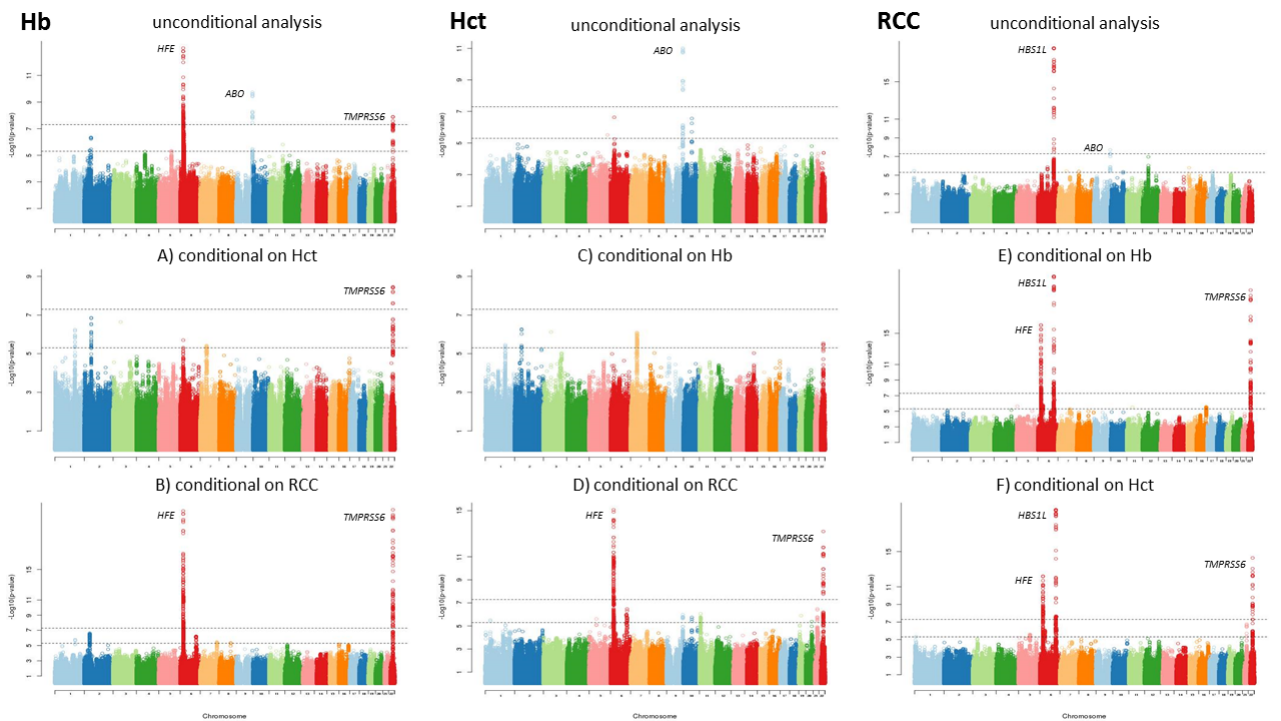
Manhattan plots of meta-analysis association results in unconditional and conditional analyses for hemoglobin (Hb), hematocrit (Hct) and red blood cell count (RCC). Results show unconditional analysis for all three traits (top row) and analysis of Hb conditional on (A) Hct and (B) RCC, of Hct conditional on (C) Hb and (D) RCC and of RCC conditional on (E) Hb and (F) Hct. Line at–log_10_(*P* value) = 5.3 represents suggestive threshold and line at–log_10_(*P* value) = 7.3 significant threshold.

In analyses conditional on RBC traits we expect to see reduction in the association signal if the trait we are conditioning on plays a role in the association. Conditional analyses of all three traits, taking each one in turn as the dependent variable with the other two as covariates, showed total loss of signal at the *ABO* locus (Fig 2). When conditioned on Hct, the signal for Hb at the *HFE* locus disappeared (Fig 2A). However, when the analysis was conditional on RCC, the signals for Hb and Hct at both *HFE* and *TMPRSS6* loci rose (Fig 2B and 2D). The same happened when the analysis for RCC was conditional on Hb and Hct, with both *HFE* and *TMPRSS6* loci becoming significant (Fig 2E and 2F). The signal at the *HBS1L* locus remained unaffected by conditional analyses (Fig 2E and 2F).

### Association of *ABO* SNPs with other cardiometabolic traits

**Fig 3:**
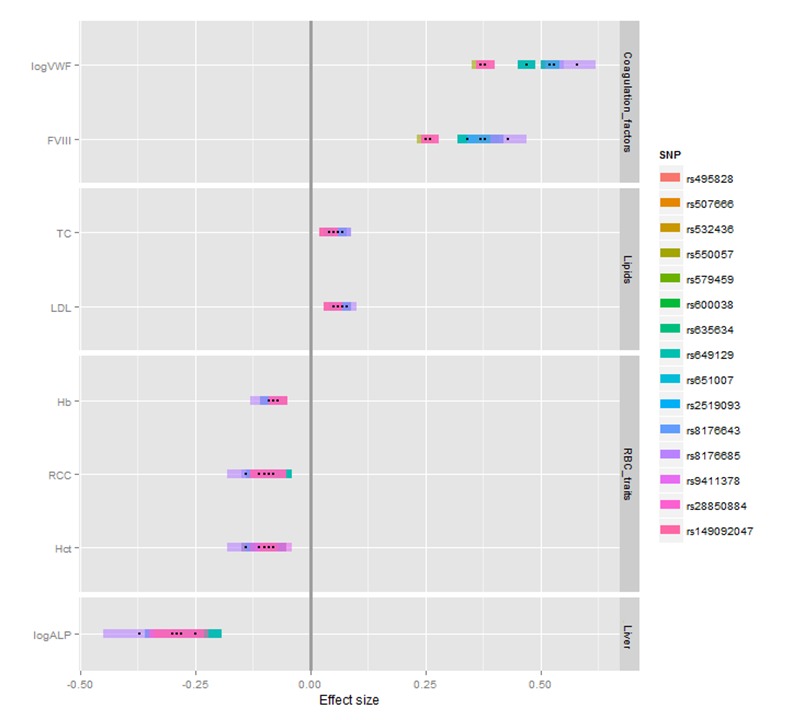
Comparison of the effect sizes on 15 *ABO* SNPs between eight different traits. Traits include von Willebrand factor (log transformed, logVWF) and factor VIII (FVIII) for coagulation factors, total cholesterol (TC) and low-density lipoprotein (LDL) for lipids, hemoglobin (Hb), red blood cell count (RCC) and hematocrit (Hct) for the red blood cell (RBC) traits and alkaline phosphatase (log transformed, logALP) for liver marker group. The colored bar for each SNP represents the 95% confidence interval of the effect size.

Due to the richly phenotyped studies within the UCLEB consortium we were able to access the results of prior large scale meta-analyses of a large number of ‘intermediate’ continuous traits, predominantly cardiometabolic traits, including lipids/lipoproteins, coagulation factors and demographic factors [25]. We identified five cardiometabolic traits that were significantly associated with the *ABO* locus (S7 Fig): factor VIII (FVIII), von Willebrand factor (log transformed, logVWF), low-density lipoprotein cholesterol (LDL), total cholesterol (TC), and alkaline phosphatase (log transformed, logALP), all of which have been previously reported to be associated with *ABO* SNPs [26–32]. The study characteristics for these traits are given in S5 Table. We directly compared the effect sizes of 15 RBC-associated *ABO* SNPs across the three RBC and five additional cardiometabolic traits in the UCLEB consortium dataset using standardized regression coefficients (β; Fig 3). Direct comparison showed variation in effect size from β = -0.30 for logALP on rs507666 to β = 0.52 on the same SNP for logVWF, and from β = -0.25 to β = 0.47 on the widely cited rs651007. The direction of the minor allele effects on RBC traits and logALP were negative for all SNPs while the same alleles had positive effects on LDL- and total-cholesterol and coagulation factors, most notably logVWF. Only FVIII showed weak correlation with some of the RBC traits, all other traits were not correlated with RBC traits.

## Discussion

Seven studies in the UCLEB Consortium with a total of 11 093 individuals of European ancestry contributed data for this meta-analysis of six red blood cell traits. There were 394 non-overlapping SNPs in five loci associated with the RBC traits at genome-wide significance level, out of which 86% were significantly associated with more than one trait. The five loci (with candidate gene) identified were: 6p22.1-6p21.33 (*HFE*), 6q23.2 (*HBS1L*), 6q23.3 (contains no genes), 9q34.3 (*ABO*) and 22q13.1 (*TMPRSS6*). Associations at the *HFE*, *HBS1L* and *TMPRSS6* genes with RBC traits are well established across different ethnic groups, adults and/or children and associated with one or more red blood cell traits [2–4, 6–10, 12, 13, 21, 33]. Both *HFE* and *TMPRSS6* are involved in iron homeostasis by regulating the production of hepcidin, which is considered to be the “master” iron regulator; mutations in *HFE* cause the most common form of hereditary hemochromatosis while mutations in *TMPRSS6* cause iron-refractory iron deficiency anemia (IRIDA). SNPs in the *HBS1L-MYB* locus are associated with fetal hemoglobin levels and all hematological traits of the three major blood-cell lineages: white blood cells, RBC and platelets. SNPs in the 6q23.3 locus show association with MCH and MCV in several studies. This locus does not contain any genes itself, however, the nearby *CITED2* gene has been reported as a candidate gene [6, 7, 9, 13]. *CITED2* inhibits transactivation of hypoxia-inducible factor 1-alpha (HIF1A)-induced genes affecting essential physiological processes, including iron homeostasis. Underlying correlation between the traits is reflected in the results. High correlation between Hb, Hct and RCC shows in the association of these traits with *ABO* locus, while high correlation between MCH and MCV shows at almost all other associated loci. Similar patterns were observed in the CHARGE Consortium [6], where it was suggested that the observed patterns characterize various clinical hematological diseases and provide context for interpretation of the results.

We have also replicated and confirmed the association of *ABO* SNPs with Hb, Hct and RCC in Europeans, previously reported in East Asians, Caucasians and African-Americans [5, 7, 9, 10, 13, 34]. By imputation to 1000 Genomes data, we identified 11 new SNPs significantly associated with these traits and replicated all but one in an independent population, further refining the associations. We then focused on the characterization of the *ABO* locus, looking to dissect its association with RBC traits by means of conditional analysis. First we investigated whether these three RBC traits were associated with the identified loci, especially *ABO*, independently. The results indicated that the signals at *HFE* and *TMPRSS6* were mediated through the MCV component of Hct and through Hb. This is in agreement with findings that patients with hereditary hemochromatosis have higher mean Hb and MCV, and lower mean RCC than controls [35, 36], while in iron-deficiency states such as IRIDA, low values for Hb and MCV occur, with RCC being higher [2, 37]. In both conditions, changes in Hb and MCV are in the opposite direction to RCC, consistent with our observations in the conditional analysis. The *HBS1L* signal for RCC appeared not to be influenced by Hb or Hct, while the signal at the *ABO* locus was influenced by all three traits. Consistent with this association of *ABO* with RBC traits, a recent study of biomarkers of iron status found that the *ABO* locus was associated with serum ferritin concentration, an indicator of body iron stores, in Europeans [38]. A study conducted in South Eastern Nigeria found a significant difference in serum iron concentration and total iron binding capacity (TIBC) between the ABO blood groups, with group A having highest and group O the lowest values for both iron parameters [39]. Given that the other four loci we identified are well accepted as having a direct or indirect role in iron homeostasis, it is an intriguing possibility that the *ABO* locus may also influence RBC traits through an effect on iron.

Further, we looked at the distribution of the traits associated with the *ABO* locus in our study according to haplotypes predicting blood group, which showed a significant difference in trait means between the blood group types. Results from analyses conditional on the genetic determinants of blood groups suggested that the locus at ~135.154 Mb is greatly influenced by the blood group O deletion. They also indicated the possibility of two loci here: one at the 5′ end at ~135.15 Mb with rs507666 as the lead SNP, and another one proximal, between ~135.15 and ~135.14 Mb, with rs28850884 as lead SNP. However, these two loci are not entirely independent–SNPs in both loci are in moderate LD with each other and the signal on both of them disappears completely in analysis conditional on rs507666 (results not shown). Since the *ABO* locus is also associated with several other traits and diseases, including lipids, coagulation factors, markers of endothelial function, cardiovascular disease, infectious disease and cancer [26–32, 40–42], we investigated whether other traits available in the UCLEB Consortium are associated with it. This directly demonstrated the pleiotropic associations of the same *ABO* SNPs with other cardiometabolic traits: coagulation factors (FVIII, logVWF), lipids (LDL, TC) and a liver function trait (logALP). This is not surprising given that, in addition to their expression on red blood cells, ABO antigens are widely expressed on different cell types, including vascular endothelium and epithelium [42, 43]. Hence they have potential for wide influence upon human traits. ABO determinants are also present in body fluids and are carried on the coagulation protein von Willebrand factor, consistent with the *ABO* locus associations we demonstrated with these traits [44]. The direction of effect on the *ABO* alleles associated with cardiometabolic traits in our study were consistent with previous reports. For different traits, these effect directions are widely reported to be associated with generally either beneficial or detrimental effects upon cardiometabolic risk. Our results show that for the 15 *ABO* SNPs most strongly associated with RBC traits, an increase in copies of the minor allele was associated with increased coagulation factor and lipid levels and decreased RBC traits and alkaline phosphatase. This effect was reversed for blood group O, resulting in decreased coagulation factor and lipid levels and increased Hb, Hct, RCC and ALP, both acting protective. These findings add to the mounting evidence for the role of the ABO blood group as a risk factor for development of cardiovascular disease, thrombosis, diabetes mellitus and cancer, with the O blood type being protective [45–49].

Studies such as ARIC [50] and HEIRS [51] showed that *HFE* C282Y homozygosity, which is associated with increased iron absorption, is also associated with lower total and LDL cholesterol. Equally, studies show that triglyceride, total cholesterol and LDL cholesterol are decreased in women with iron deficiency anemia [52, 53]. Both conditions have in common the diminished production of hepcidin, which in turn leads to iron-depleted macrophages which may negatively regulate systemic cholesterol levels and so offer protection from atherosclerosis [54]. Evidence from mouse studies suggests that macrophage iron depletion may have pro-inflammatory effects [55]; reviewed in [56], which could be relevant to O blood group individuals as discussed above. rs651007, close to the 5′ end of the *ABO* gene and significantly associated with RBC traits in our study, was the only SNP associated with decreased ferritin concentration and decreased atherosclerosis risk in a recent study of the role of iron and hepcidin in atherosclerosis [57]. Additionally, increased levels of IL-10 have been found in acute coronary syndrome cases compared to healthy controls, reflecting an underlying inflammatory state, with levels of IL-10 being higher in blood type O compared to individuals with other blood types and associated with poor outcome in these patients [58]. They suggested that inflammation is a more important risk factor in these individuals, while increased coagulation is more important in non-O blood type [58].

Finally, the current discovery analysis is somewhat biased towards loci already known to be associated with cardiometabolic disease due to the design of Metabochip [59]. Imputation to the 1000 Genomes expanded the regions covered by the chip but could not completely fill the gaps. Analysis based on GWAS coverage imputed to the latest 1000 Genomes phase could discover new loci associated with these traits. Most of the studies, including ours, focus on classic ABO blood group phenotypes. The complexity of the ABO blood group system extends to a number of different subtypes and the way that they may influence the traits and disease development requires more detailed investigation. However, we were able to further dissect the association between *ABO* locus and RBC traits as well as show how the same SNPs influence range of traits and put it in context of cardiometabolic risk. Our results support the suggestion by Johansson *et al*. [58] that the ABO blood group of an individual may determine which risk factors are more important for them.

## Materials and Methods

### Studies and phenotypes

The UCL-LSHTM-Edinburgh-Bristol (UCLEB) Consortium has been described in detail in Shah *et al*. [25]. Six RBC traits were analyzed: Hb, Hct, MCH, MCHC, MCV and RCC. We calculated missing traits as detailed in S1 Note. The total sample size in seven eligible studies was 11 093. The Cohorte Lausannoise [60] (CoLaus, N = 2848) study was approached for replication of the results due to the availability of both genotypes imputed to 1000 Genomes and the traits of interest. Discovery and replication study design are further explained in S1 Note. All participants were of European ancestry.

### Ethics Statement

Each study of the UCLEB consortium was approved by the appropriate local research ethics committee and all participants gave written informed consent, with details as follow: **1958BC**—Written consent was obtained from participants for the use of information in medical studies. The 45-year biomedical survey and genetic studies were approved by the South-East Multi-Centre Research Ethics Committee (ref: 01/1/44) and the joint UCL/UCLH Committees on the Ethics of Human Research (Ref: 08/H0714/40); **BRHS**—The National Research Ethics Service Committee for London provided ethical approval. Participants provided a written informed consent for the investigation, which was performed in accordance with the Declaration of Helsinki; **BWHHS**—Ethics approval was granted for the British Women’s Heart & Health Studies from the London Multi Centre Research Ethics Committee and 23 Local Research Ethics Committees (Harrogate: Harrogate Health Care, Shrewsbury: Shropshire Health Authority, Lowestoft: Great Yarmouth James Pagent Healthcare NHS Trust, Mansfield: North Nottinghamshire Health, Southport: North Sefton Research Ethics Committee, Merthyr Tydfil: Awdurdod Lechyd Bro Taf Health Authority, Guildford: South West Surrey Local Research Ethics Committee, Burnley: East Lancashire Health Authority, Newcastle-under-Lyme: North Staffordshire Health Authority, Exeter: Exeter Research Ethics Committee, Falkirk: Fife Health Board, Ipswich: East Suffolk Local Research Ethics Committee, Gloucester: Southmead Health Services, Carlisle: East Cumbria Local Research Ethics Committee, Dunfermline: Fife Health Board, Darlington: County Durham Local Research Ethics Committee, Ayr: Fife Health Board, Grimsby: South Humber Health Authority, Bedford: North Bedfordshire Health Authority, Wigan: Wigan & Leigh LREC, Scunthorpe: South Humber Health Authority, Hartlepool: Hartlepool Local Research Ethics Committee, Bristol: Southmead Health Services). Participants were asked for written informed consent to review their medical records and for permission to perform anonymized genetic tests; **CaPS**—Ethics approval was obtained from the South East Wales Local Research Ethics Committee, and each subject signed their agreement to be involved; **EAS**—Approval for the study was given by the Lothian Health Board ethics committee and written informed consent was obtained from each subject; **ELSA**—Ethical approval for ELSA was given by the National Research Ethics Service and all participants gave written consent; **ET2DS**—Ethical permission was obtained from the Lothian Medical Research Ethics Committee. All subjects attended a dedicated research clinic where they gave written informed consent for participation in the study; **MRC NSHD**—Ethical approval was obtained from the Central Manchester Research Ethics Committee (07/H1008/168 and 07/H1008/245) and North Thames Multi-Centre Research Ethics Committee (MREC 98/1/121). Informed written consent was obtained from the study members; **WHII**—In Whitehall II, participants provided their written informed consent and the National Health Services (NHS), Health Research Authority, National Research Ethics Service (NRES) Committee London—Harrow approved the consent procedure.

Blood samples were collected and analyzed using standard methods and assays.

For the replication study, the Institutional Review Board of the Centre Hospitalier Universitaire Vaudois (CHUV) in Lausanne and the Cantonal Ethics Committee approved the **CoLaus** study protocol and signed informed consent was obtained from participants. Starting in 2009 all participants were invited for a follow-up visit five years after the initial study, completed in 2012. This follow-up study was approved by the local ethics committee.

### Genotypes and imputation

All studies with RBC traits were genotyped using Metabochip and imputed to the 1000 Genomes (Phase1, CEU haplotype set). Genotyping, quality control (QC) procedures and imputation were performed in each study separately using the same protocol [25]. The exclusion criteria for samples and SNPs consisted of an Illumina GenCall score < 0.15, sample and SNP rates < 0.95, HWE *P* value ≤ 0.001, replicate discordance, discrepancy in sex, ethnicity and relatedness checks or against previous genotype data, where available. No MAF cut-off was applied at this stage. Imputation was done on cleaned genotypes in three phases—chunking, phasing and imputing—using the MACH1 and Minimac software [61, 62] and applying the strategy described at Minimac:1000 Genomes Imputation Cookbook. The resulting file contained ~4.5 million autosomal SNPs (R^2^ > 0.3, MAF > 0.001). The data were collated in posterior probabilities from genotype dosage, incorporating imputation uncertainty, to be used by the R Package snpStats.

### Association and meta-analysis

Based on descriptive statistics and visual inspection (Table 1; S1 Fig), association testing was done on untransformed data, separately for each study and on each trait. The associations between genotyped and imputed SNPs and traits were tested by linear regression using an additive genetic model. Adjustment was made for age, sex and diabetes status, where appropriate. Analyses were carried out using snpStats. Prior to meta-analysis, SNPs with MAF frequency < 0.005 were filtered out. Genomic-control inflation factors (λ_GC_) were calculated and used to correct test statistics from each study for each trait in the meta-analysis. Study-specific estimates of effect size were combined by fixed effects meta-analysis weighted by inverse variance using the METAL program. Genomic-control inflation factors were calculated again after the meta-analysis, but the results were not further corrected. Significance threshold was set at *P* < 5×10^−8^, suggestive at *P* < 5×10^−6^. Locus (significant or suggestive) was defined as all SNPs below the threshold with SNPs located less than 500 kb apart. LocusZoom [63] was used to display results, using 1000 Genomes Phase 1 CEU haplotype set for linkage disequilibrium (LD) calculation. The positions of SNPs are given on human genome build 19.

### Conditional analyses

Conditional analysis was performed using the SNPs encoding the two major *HFE* mutations, C282Y (rs1800562) and H63D (rs1799945), as covariates to further investigate if the signal at this locus for Hb, MCH and MCV was due to *HFE* alone. Also, to further dissect the signal at the *ABO* locus, we used analyses conditional on the genetic determinants of blood groups (AA, AO, B, AB and O) with a blood group entered as a binary variable one at the time (e.g. O vs all other groups). The three SNP haplotypes used to derive blood groups are presented in Table 4. To investigate whether the signal in the *ABO* locus for Hb, Hct and RCC affects a trait independently of the other two traits, the subset of five studies which had data on all three traits was analyzed. For a given trait, the other two traits were included in the model as covariates one at a time [64].

**Table 4:** Blood groups derived based on three SNP haplotypes.

**Blood Group**	**rs8176719**	**rs8176746**	**rs8176747**	**Frequency in UCLEB**
**AA**	G/G	C/C	G/G	8.0%
**AO**	Del/G	C/C	G/G	37.3%
**BB**	G/G	A/A	C/C	0.4%
**BO**	Del/G	A/A	C/C	8.4%
**AB**	G/G	A/C	C/G	3.5%
**O**	Del/Del			42.3%

### Association with other traits within UCLEB

To assess pleiotropy by direct comparison within UCLEB, we ran an association study on standardized phenotypes (mean = 0, SD = 1). Effect sizes (standardized regression coefficients, β) were visualized using phenotypicForest 0.2.

Pearson's *r* was calculated to assess correlation between all the traits within the study.

URLs

Minimac: 1000 Genomes Imputation Cookbook, http://genome.sph.umich.edu/wiki/Minimac:_1000_Genomes_Imputation_Cookbook

snpStats, http://www.bioconductor.org/packages/2.10/bioc/html/snpStats.html

METAL, http://www.sph.umich.edu/csg/abecasis/Metal/

phenotypicForest 0.2, http://chrisladroue.com/phorest/

European Genome-phenome Archive, http://www.ebi.ac.uk/ega

## Supporting Information

S1 Fig Histograms for six red blood cell traits. Please note that the hemoglobin values for WHII were rounded.
(TIF)

S2 Fig Manhattan plots of meta-analysis association results for red blood cell traits in the UCLEB consortium. Line at–log_10_(*P* value) = 5.3 represents suggestive threshold and line at–log_10_(*P* value) = 7.3 significant threshold.
(TIF)

S3 Fig Q-Q plots of meta-analysis association results for red blood cell traits in the UCLEB consortium. (TIF)

S4 Fig Venn diagram of overlap between loci above the significance threshold (*P* < 5×10^−8^). Traits include hemoglobin (Hb), hematocrit (Hct), mean corpuscular hemoglobin (MCH), mean corpuscular hemoglobin concentration (MCHC), mean corpuscular volume (MCV) and red blood cell count (RCC).
(TIF)

S5 Fig Manhattan plots of meta-analysis association results for Hb, MCH and MCV in analyses conditional on two major *HFE* gene mutations, C282Y and H63D. Line at–log_10_(*P* value) = 5.3 represents suggestive threshold and line at–log_10_(*P* value) = 7.3 significant threshold.
(TIF)

S6 Fig Comparison between van der Harst et al. and our study on overlapping 79 SNPs within +/- 10Kb from *ABO* gene. Regional plots for (A-B) Hb, (D-E) Hct and (E-F) RCC show two loci: (A,C,E) first locus at ~136.154 close to the 5′ region of the gene and (B,D,F) second locus at ~136.131 Mb, using 1000 genomes (Phase 1, EUR haplotype set) for LD calculation. Blue line represents suggestive and red line significant threshold.
(TIF)

S7 Fig Meta-analysis association results for three red blood cell and five cardiometabolic traits in the *ABO* locus. Regional plots show association results for (A) von Willebrand factor (log transformed, logVWF), (B) factor VIII (FVIII), (C) total cholesterol (TC), (D) low-density lipoprotein (LDL), (E) hemoglobin (Hb), (F) red blood cell count (RCC), (G) hematocrit (Hct) and (H) alkaline phosphatase (log transformed, logALP).The most significant associations for logVWF are capped at 1×10^−325^. The most significant SNP for Hb, rs507666, is highlighted throughout to facilitate comparison of results. Blue line represents suggestive and red line significant threshold.
(TIF)

S1 Note Supplementary note. (DOCX)

S2 Note Data access arrangements. (DOCX)

S1 Table Formulae for calculating missing erythrocyte traits. (DOCX)

S2 Table Genomic-control inflation factors (λ_GC_) for each study and trait. λ_GC_ were recalculated for each trait after meta-analysis.
(DOCX)

S3 Table Meta-analysis results. All SNPs significantly associated with one or more red blood cell traits (*P* < 5×10^−8^) in seven studies from the UCLEB consortium (separate file).
(XLSX)

S4 Table Results of replication in the CoLaus study. Results for 47 SNPs in/around *ABO* gene, which were either significantly or suggestively associated with Hb, Hct and RCC in seven studies from the UCLEB consortium. The rs numbers of the SNPs which were significantly associated with Hb, Hct and/or RCC (*P* < 5×10^−8^, N = 15) in the discovery study are highlighted in bold and the corresponding fields are marked in blue; these are additionally shown in Table 3. The significant replication results (*P* < 0.05) are marked in red (separate file).
(XLSX)

S5 Table Study characteristics on other cardiometabolic traits associated with *ABO* SNPs and available in UCLEB. (DOCX)

S6 Table Pearson’s *r* correlation coefficient between six red blood cell traits and five cardiometabolic traits. (XLSX)
