# Correction: Replication and Characterization of Association between *ABO* SNPs and Red Blood Cell Traits by Meta-Analysis in Europeans

**DOI:** 10.1371/journal.pone.0193627

**Published:** 2018-02-23

**Authors:** The PLOS ONE Staff

The author list appears incorrectly. The publisher apologizes for the error. The correct author list is as follows: Stela McLachlan, Claudia Giambartolomei, Jon White, Pimphen Charoen, Andrew Wong, Chris Finan, Jorgen Engmann, Tina Shah, Micha Hersch, Clara Podmore, Alana Cavadino, Barbara J. Jefferis, Caroline E. Dale, Elina Hypponen, Richard W. Morris, Juan P. Casas, Meena Kumari, Yoav Ben-Shlomo, Tom R. Gaunt, Fotios Drenos, Claudia Langenberg, Diana Kuh, Mika Kivimaki, Rico Rueedi, Gerard Waeber, Aroon D. Hingorani, Jacqueline F. Price, Ann P. Walker, on behalf of the UCLEB Consortium.
