# Supplementary figures and images for: Replication and Characterization of Association between ABO SNPs and Red Blood Cell Traits by Meta-Analysis in Europeans
Source: PLoS One. 2016 Jun 9;11(6):e0156914. doi: 10.1371/journal.pone.0156914 (PMC4900668; doi:10.1371/journal.pone.0156914)

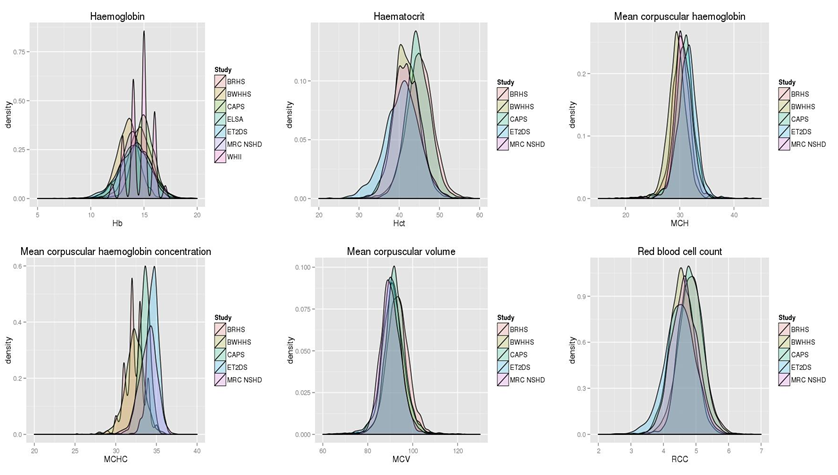

Supplement: S1 Fig — Please note that the hemoglobin values for WHII were rounded. (TIF) [file pone.0156914.s001.tif]

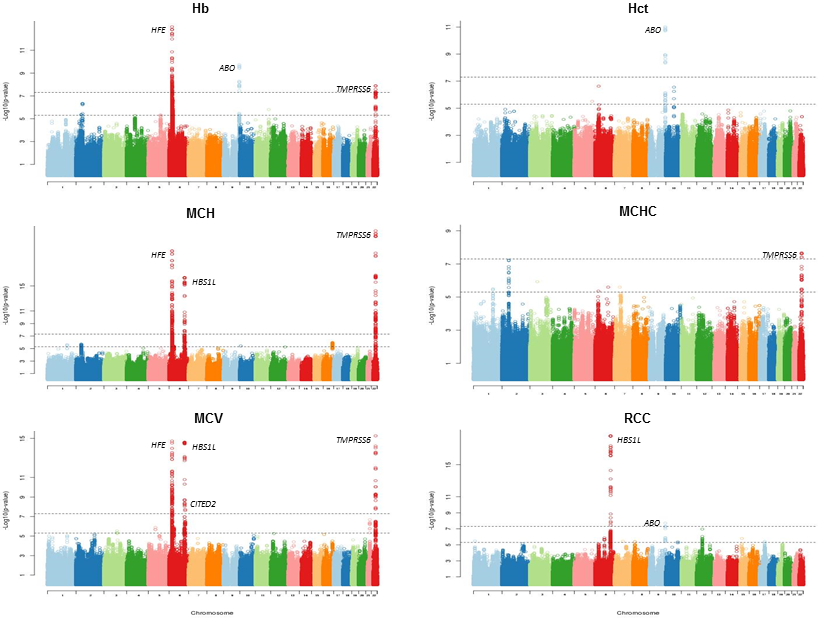

Supplement: S2 Fig — Line at–log10(P value) = 5.3 represents suggestive threshold and line at–log10(P value) = 7.3 significant threshold. (TIF) [file pone.0156914.s002.tif]

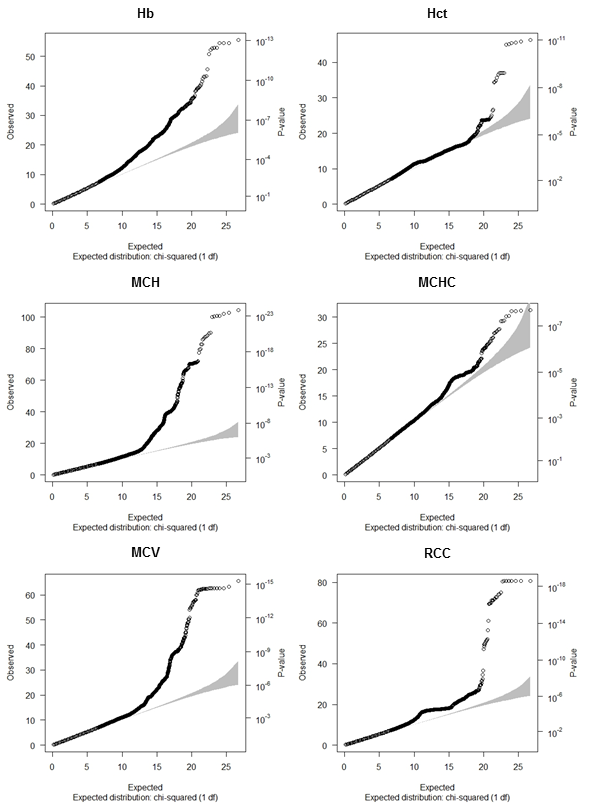

Supplement: S3 Fig — (TIF) [file pone.0156914.s003.tif]

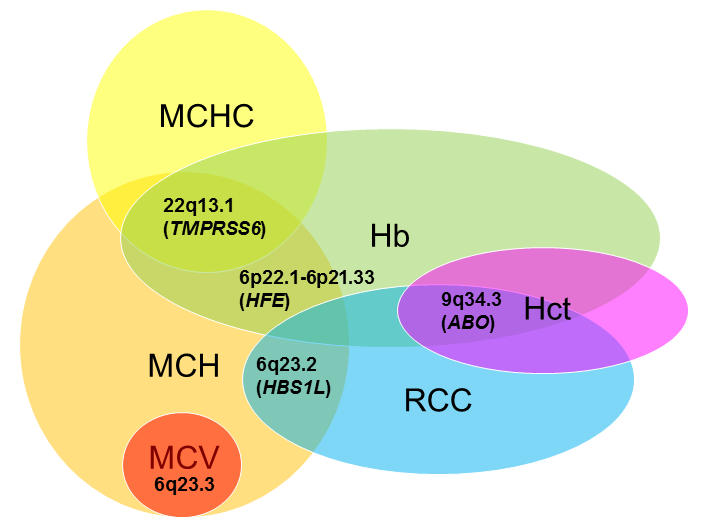

Supplement: S4 Fig — Traits include hemoglobin (Hb), hematocrit (Hct), mean corpuscular hemoglobin (MCH), mean corpuscular hemoglobin concentration (MCHC), mean corpuscular volume (MCV) and red blood cell count (RCC). (TIF) [file pone.0156914.s004.tif]

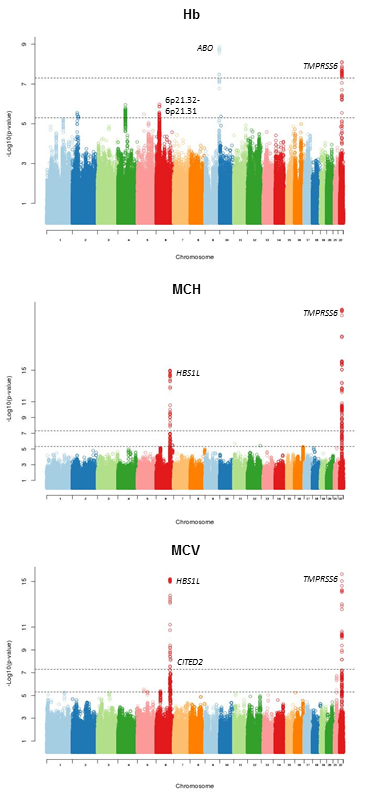

Supplement: S5 Fig — Line at–log10(P value) = 5.3 represents suggestive threshold and line at–log10(P value) = 7.3 significant threshold. (TIF) [file pone.0156914.s005.tif]

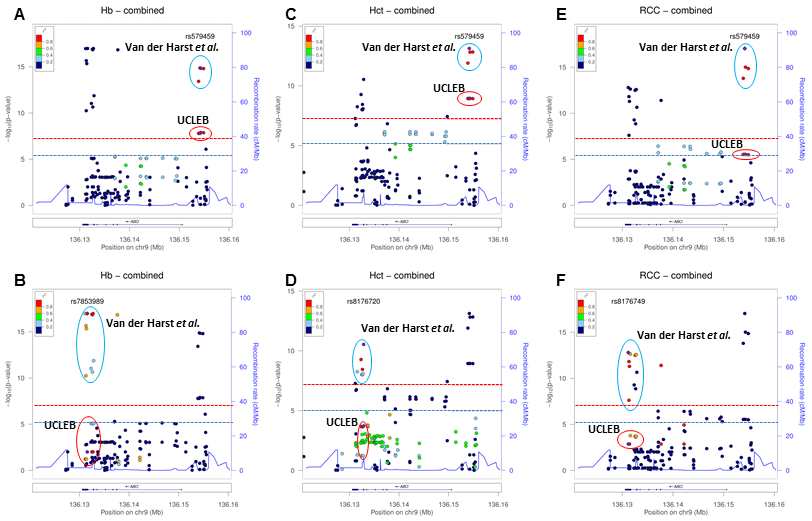

Supplement: S6 Fig — Regional plots for (A-B) Hb, (D-E) Hct and (E-F) RCC show two loci: (A,C,E) first locus at ~136.154 close to the 5′ region of the gene and (B,D,F) second locus at ~136.131 Mb, using 1000 genomes (Phase 1, EUR haplotype set) for LD calculation. Blue line represents suggestive and red line significant threshold. (TIF) [file pone.0156914.s006.tif]

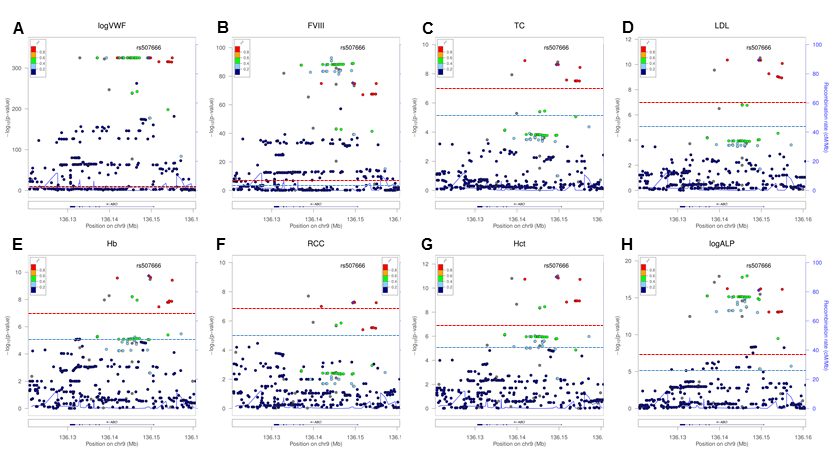

Supplement: S7 Fig — Regional plots show association results for (A) von Willebrand factor (log transformed, logVWF), (B) factor VIII (FVIII), (C) total cholesterol (TC), (D) low-density lipoprotein (LDL), (E) hemoglobin (Hb), (F) red blood cell count (RCC), (G) hematocrit (Hct) and (H) alkaline phosphatase (log transformed, logALP).The most significant associations for logVWF are capped at 1×10−325. The most significant SNP for Hb, rs507666, is highlighted throughout to facilitate comparison of results. Blue line represents suggestive and red line significant threshold. (TIF) [file pone.0156914.s007.tif]
