# Supplementary material for: Replication and Characterization of Association between ABO SNPs and Red Blood Cell Traits by Meta-Analysis in Europeans
Source: PLoS One. 2016 Jun 9;11(6):e0156914. doi: 10.1371/journal.pone.0156914 (PMC4900668; doi:10.1371/journal.pone.0156914)
Supplement: S1 Note — (DOCX) [file pone.0156914.s008.docx]

**S1 Note. Supplementary Note.**

**Abbreviations for traits:** Hb, hemoglobin; Hct, hematocrit; MCH, mean corpuscular hemoglobin; MCHC, mean corpuscular hemoglobin concentration; MCV, mean corpuscular volume; RCC, red blood cell count; FVIII, factor VIII; logVWF, log transformed von Willebrand factor; LDL, low-density lipoprotein cholesterol; TC, total cholesterol; logALP, log transformed alkaline phosphatase.

**Abbreviations for studies:** 1958BC, 1958 Birth Cohort; BRHS, British Regional Heart Study; CaPS, Caerphilly Prospective Study; EAS, Edinburgh Artery Study; ELSA, English Longitudinal Study of Ageing; ET2DS, Edinburgh Type 2 Diabetes Study, MRC NSHD, MRC National Survey of Health and Development; WHII, Whitehall II Study.

**Discovery cohort**

The UCL-LSHTM-Edinburgh-Bristol (UCLEB) Consortium has been described in detail in Shah *et al*.(1). Within the UCLEB consortium, seven studies had information on at least one red blood cell trait: British Regional Heart Study(2) (BRHS, N = 2310), British Women’s Heart and Health Study(3) (BWHHS; N = 1884), Caerphilly Prospective Study(4), (CaPS; N = 1268), English Longitudinal Study of Ageing(5) (ELSA; N = 1881), Edinburgh Type 2 Diabetes Study(6) (ET2DS; N = 996), MRC National Survey of Health and Development(7, 8) (MRC NSHD; N = 1672) and Whitehall II Study(9) (WHII, N = 1082). Each study was approved by the appropriate local research ethics committee and all participants gave informed consent. Blood samples were collected and analyzed using standard methods and assays.

ELSA and WHII had only Hb available. BWHHS and CaPS had MCHC missing completely and ET2DS had both MCH and MCHC missing. MCHC was not calculated for 419 participants in MRC NSHD.

**Calculating missing traits**

MCH and MCHC are usually calculated from other RBC trait measurements (using the formulae provided in Supplementary Table S1), and these were calculated where missing. To check the validity of this approach, we first recalculated these traits in the studies where data provided directly from the laboratories was available. The differences between supplied and calculated MCH (pg) and MCHC (g/dL) were normally distributed in each study with means (SDs) of 0.0019 (0.08) in BRHS, -0.0006 (0.07) in BWHHS, 0.0239 (0.07) in CaPS and -0.0011 (0.08) in MRC NSHD, and 0.0043 (0.21; larger since the provided MCHC were rounded to whole number) in BRHS and 0.0060 (0.08) in a subset of MRC NSHD with information available, respectively. Based on these results, we concluded that the difference between supplied and calculated data was negligible (due to rounding), and therefore proceeded to include calculated MCH and MCHC values from studies where this information was otherwise not available.

**Covariates for the association analysis**

As described in “Methods”, adjustment was made for age, sex and diabetes status, where appropriate. However, data on diabetes status in the MRC NSHD was from the wave of data collection preceding that for RBC traits, while in WHII, diabetes status was not available from a time point close to that of Hb measurement, so was not included as a covariate in the analysis. Meta-analysis for Hb omitting the WHII study (for which relevant diabetes status was unavailable) and further adjustment for smoking status and estimated glomerular filtration rate did not change the significant signals (results not shown).

**Replication cohort**

The design of the Cohorte Lausannoise (CoLaus) study has been described previously(10). Briefly, this is a population-based study conducted between 2003 and 2006, which recruited over 6000 subjects in Lausanne, Switzerland. The following inclusion criteria were applied: (a) voluntary participation in the examination, including blood sample, (b) aged 35-75 years, and (c) Caucasian origin, defined as both parents and grand-parents being Caucasian (as determined by birth place). The Institutional Review Board of the Centre Hospitalier Universitaire Vaudois (CHUV) in Lausanne and the Cantonal Ethics Committee approved the study protocol and signed informed consent was obtained from participants. Starting in 2009 all participants were invited for a follow-up visit five years after the initial study, completed in 2012. This follow-up study was approved by the local ethics committee. During the follow-up visit, variables similar to those measured in the cross-sectional study were determined, with the addition of a complete blood count. The latter was measured on a haematology Sysmex XE2100analyser (TOA Medical Electronics, Kobe, Japan) according to the manufacturer’s indications.

**Heterogeneity in MCH results**

Most of the significant results with high I2 values (34 out of 38) are for MCH in two loci only: 6q23.2 and 22q13.1 (Supplementary Table S3: “HetPVal<0.05”). We speculated that this observed heterogeneity could be a consequence of including calculated MCH values for one study only. However, a repeat analysis that included only data provided directly from the laboratories did not resolve the issue of heterogeneity (data not shown). Since these SNPs are in loci known to be associated with MCH, as described above, we made no further attempts to identify the cause of heterogeneity.

**Comparison with published studies**

To compare our results with the latest published study, we accessed summary statistics from the genome-wide association study described by van der Harst *et al*.(11), which are available from the European Genome–phenome Archive ([EGA](http://www.ebi.ac.uk/ega)) under accession number EGAS00000000132. There were 79 SNPs within +/- 10 Kb of the *ABO* gene in common between van der Harst *et al*.(11) and the present study. The *P* values from both studies were plotted to compare the results within the *ABO* locus (S6 Fig). Van der Harst *et al*.(11) reported the possibility of two independent loci within the *ABO* locus for Hb and RCC, as ascertained by conditional analysis. They reported rs579459 as the lead SNP for Hb at the first locus, at ~136.154 Mb, close to the 5′ region of *ABO* gene. This SNP was significant in both studies (S6A Fig). The second locus, at ~136.131 Mb, included SNPs within the *ABO* gene, with rs7853989 as the lead SNP, however, this locus was not significant in the present study (S6B Fig). A similar pattern was seen for Hct (S6C-D Fig), although van der Harst *et al*.(11) didn’t report a second locus for this trait, and RCC (S6E-F Fig). MAF for all examined SNPs was similar between the studies, except on rs8176747: 9% in van der Harst *et al*.(11) vs 6% in the present study. Also, maximum sample size was 61 153 for Hb, 53 087 for Hct and 53 659 for RCC in van der Harst *et al*.(11) compared to 11 093 for Hb and 8129 Hct and RCC, respectively, in the present study. In summary, we could not confirm the presence of two independent loci within *ABO* locus as suggested by van der Harst *et al*.(11) in our unconditional analysis. The first locus at ~136.154 Mb was significant in both studies but the second locus at ~136.131 Mb was not significant in our study. However, in the analysis conditional on blood group O, the second locus showed a *P* value below the suggestive threshold (Fig 1B). Pairwise LD calculations (available on a subset of SNPs from 1000 Genomes, Phase 3, CEU haplotype set) show that the most significant SNPs from the second locus are in strong LD (r^2^=0.92-1) with rs8176647 and rs8176646 (A/B blood type). In contrast, seven SNPs from the first locus are in moderate LD (r^2^=0.48) with rs8176719, which codes for O blood type.

**Attempt to link the SNPs to genes based on functional annotation**

In order to identify the candidate gene in the 6q23.3 locus, we have looked up 2 SNPs in this locus significantly associated with MCV and 25 SNPs suggestively associated with MCV and MCH using freely available gene expression datasets. We did not find evidence of the 27 SNPs influencing expression in cis. We investigated whether the 27 SNPs were among the significant cis-eQTLs among the 44 tissues reported in the Genotype-tissue Expression dataset version 6 (<http://www.ncbi.nlm.nih.gov/pubmed/23715323>) available from the web-portal (<http://www.gtexportal.org/home/datasets>) and among the liver expression dataset from 960 human liver samples (<http://www.nature.com/ng/journal/v44/n5/full/ng.2248.html>).

**References**

1. Shah T, Engmann J, Dale C, Shah S, White J, Giambartolomei C, et al. Population genomics of cardiometabolic traits: design of the University College London-London School of Hygiene and Tropical Medicine-Edinburgh-Bristol (UCLEB) Consortium. PloS one. 2013;8(8):e71345. 10.1371/journal.pone.0071345.

2. Shaper AG, Pocock SJ, Walker M, Cohen NM, Wale CJ, Thomson AG. British Regional Heart Study: cardiovascular risk factors in middle-aged men in 24 towns. British medical journal. 1981;283(6285):179-86.

3. Lawlor DA, Bedford C, Taylor M, Ebrahim S. Geographical variation in cardiovascular disease, risk factors, and their control in older women: British Women's Heart and Health Study. Journal of epidemiology and community health. 2003;57(2):134-40.

4. Bainton D, Miller NE, Bolton CH, Yarnell JW, Sweetnam PM, Baker IA, et al. Plasma triglyceride and high density lipoprotein cholesterol as predictors of ischaemic heart disease in British men. The Caerphilly and Speedwell Collaborative Heart Disease Studies. British heart journal. 1992;68(1):60-6.

5. Marmot M, Banks J, Blundell R, Lessof C, Nazroo J. Health, Wealth and Lifestyles of the Older Population in England: The 2002 English Longitudinal Study of Ageing. London: Institute for Fiscal Studies. 2003.

6. Price JF, Reynolds RM, Mitchell RJ, Williamson RM, Fowkes FG, Deary IJ, et al. The Edinburgh Type 2 Diabetes Study: study protocol. BMC endocrine disorders. 2008;8:18.

7. Wadsworth M, Kuh D, Richards M, Hardy R. Cohort Profile: The 1946 National Birth Cohort (MRC National Survey of Health and Development). International journal of epidemiology. 2006;35(1):49-54.

8. Kuh D, Pierce M, Adams J, Deanfield J, Ekelund U, Friberg P, et al. Cohort profile: updating the cohort profile for the MRC National Survey of Health and Development: a new clinic-based data collection for ageing research. International journal of epidemiology. 2011;40(1):e1-9.

9. Marmot MG, Smith GD, Stansfeld S, Patel C, North F, Head J, et al. Health inequalities among British civil servants: the Whitehall II study. Lancet. 1991;337(8754):1387-93.

10. Firmann M, Mayor V, Vidal PM, Bochud M, Pecoud A, Hayoz D, et al. The CoLaus study: a population-based study to investigate the epidemiology and genetic determinants of cardiovascular risk factors and metabolic syndrome. BMC cardiovascular disorders. 2008;8:6.

11. van der Harst P, Zhang W, Mateo Leach I, Rendon A, Verweij N, Sehmi J, et al. Seventy-five genetic loci influencing the human red blood cell. Nature. 2012;492(7429):369-75.
