# Supplementary material for: Replication and Characterization of Association between ABO SNPs and Red Blood Cell Traits by Meta-Analysis in Europeans
Source: PLoS One. 2016 Jun 9;11(6):e0156914. doi: 10.1371/journal.pone.0156914 (PMC4900668; doi:10.1371/journal.pone.0156914)
Supplement: S2 Note — (DOCX) [file pone.0156914.s009.docx]

**S2 Note. Data access arrangements.**

Summary statistics for all SNPs used in the analysis is available to researchers upon request, subject to approval. Data request form can be obtained by emailing Tina Shah at [t.shah@ucl.ac.uk](mailto:t.shah@ucl.ac.uk).

Data access arrangements for individual contributing studies are as follows:

**1958BC**

The 1958 birth cohort data can be accessed via the UK Data Service (<http://ukdataservice.ac.uk/>).

**BRHS**

The collection and management of data over the last 34 years of the BRHS has been made possible through grant funding from UK government agencies and charities. We welcome proposals for collaborative projects and data sharing (http://www.ucl.ac.uk/pcph/research-groups-themes/brhs-pub). For general data sharing enquiries, please contact Lucy Lennon ([l.lennon@ucl.ac.uk](mailto:l.lennon@ucl.ac.uk)).

**BWHHS**

All BWHHS data collected is held by the research team based at London School of Hygiene and Tropical Medicine, for ongoing analysis. If you would like to collaborate with the BWHHS team, contact the study coordinator, Antoinette Amuzu (antoinette.amuzu@lshtm.ac.uk) Data and biological samples provided to the collaborators can only be used for the purposes originally stated and must not be used in any other way without re-application to the BWHHS team. No data should be passed on to any third party unless they were specified in the original application.

**CaPS**

Data used for the Caerphilly Prospective study (CaPS) was made available by the CaPS access committee. More information about its managed access procedure is available on the study website (<http://www.bris.ac.uk/social-community-medicine/projects/caerphilly/collaboration/>).

**CoLaus**

Data from the CoLaus/PsyCoLaus study can be requested according to the procedure described on the CoLaus website (<http://www.colaus.ch/en/cls_home/cls_pro_home/cls-research-3.htm>).

**ELSA**

ELSA data are made available through the ESDS website (<http://www.elsa-project.ac.uk/availableData>).

**EAS and** **ET2DS**

Edinburgh Artery Study and Edinburgh Type 2 Diabetes Study data are available to researchers upon request, subject to approval by the data sharing committee. Data request forms can be obtained by emailing Stela McLachlan ([stela.mclachlan@ed.ac.uk](mailto:stela.mclachlan@ed.ac.uk)).

**MRC NSHD**

The NSHD data are made available to researchers who submit data requests ([mrclha.swiftinfo@ucl.ac.uk](mailto:mrclha.swiftinfo@ucl.ac.uk)). More information is available in the full policy documents (<http://www.nshd.mrc.ac.uk/data.aspx>). Managed access is in place for this study to ensure that use of the data are within the bounds of consent given previously by participants, and to safeguard any potential threat to anonymity since the participants are all born in the same week.

**Whitehall II**

Data from the Whitehall II study are made publicly available as described in the Whitehall II data sharing policy (<https://www.ucl.ac.uk/whitehallII/data-sharing>).
