# Supplementary material for: Replication and Characterization of Association between ABO SNPs and Red Blood Cell Traits by Meta-Analysis in Europeans
Source: PLoS One. 2016 Jun 9;11(6):e0156914. doi: 10.1371/journal.pone.0156914 (PMC4900668; doi:10.1371/journal.pone.0156914)
Supplement: S1 Table — (DOCX) [file pone.0156914.s010.docx]

**Supplementary Table S1:** **Formulae for calculating missing erythrocyte traits.**

| Trait | Formula | Units |
| --- | --- | --- |
| MCH | [Hb (g/dL) / RCC (×10^12^/L)]×10 | pg/cell |
| MCHC | [MCH (pg/cell) / MCV (fL)]×100 | g/dL |
