# Supplementary material for: Replication and Characterization of Association between ABO SNPs and Red Blood Cell Traits by Meta-Analysis in Europeans
Source: PLoS One. 2016 Jun 9;11(6):e0156914. doi: 10.1371/journal.pone.0156914 (PMC4900668; doi:10.1371/journal.pone.0156914)
Supplement: S2 Table — λGC were recalculated for each trait after meta-analysis. (DOCX) [file pone.0156914.s011.docx]

**Supplementary Table S2:** **Genomic-control inflation factors (λ_GC_) for each study and trait.** λ_GC_ were recalculated for each trait after meta-analysis. Calculated traits are marked in red.

|  | Hb | Hct | MCH | MCHC | MCV | RCC |
| --- | --- | --- | --- | --- | --- | --- |
| BRHS | 1.022 | 1.013 | 1.007 | 0.998 | 1.004 | 1.023 |
| BWHHS | 1.012 | 1.015 | 0.990 | 1.005 | 1.007 | 1.041 |
| CaPS | 0.991 | 1.006 | 0.999 | 0.976 | 0.994 | 1.027 |
| ELSA | 1.012 |  |  |  |  |  |
| ET2DS | 1.014 | 1.015 | 1.007 | 0.996 | 1.010 | 1.001 |
| MRC NSHD | 0.987 | 0.994 | 1.022 | 1.005 | 1.015 | 0.996 |
| WHII | 1.005 |  |  |  |  |  |
| Meta-analysis | 1.048 | 1.038 | 1.048 | 0.995 | 1.053 | 1.068 |
