# Supplementary material for: Replication and Characterization of Association between ABO SNPs and Red Blood Cell Traits by Meta-Analysis in Europeans
Source: PLoS One. 2016 Jun 9;11(6):e0156914. doi: 10.1371/journal.pone.0156914 (PMC4900668; doi:10.1371/journal.pone.0156914)
Supplement: S5 Table — (DOCX) [file pone.0156914.s014.docx]

**Supplementary Table S5: Study characteristics on other cardiometabolic traits associated with *ABO* SNPs and available in UCLEB.**

| Study | F, % | Age, year | N | FVIII,  iu/dL | N | logVWF | N | LDL,  mmol/L | N | TC,  mmol/L | N | logALP |
| --- | --- | --- | --- | --- | --- | --- | --- | --- | --- | --- | --- | --- |
| 1958BC | 45 |  |  |  | 5620 | 4.76 (0.33) | 5356 | 3.44 (0.91) | 5687 | 5.90 (1.08) |  |  |
| BRHS | 0 | 68.91 (5.62) | 2338 | 133.42 (32.12) | 2341 | 4.90 (0.34) | 2277 | 3.89 (1.00) | 2331 | 6.37 (1.04) |  |  |
| BWHHS | 100 | 70.75 (5.31) | 1941 | 165.35 (38.96) | 1942 | 4.98 (0.32) | 1880 | 4.14 (1.10) | 1933 | 6.63 (1.23) |  |  |
| CaPS | 0 |  | 935 | 97.85 (30.78) | 1197 | 4.71 (0.35) | 1240 | 3.73 (0.89) | 1277 | 5.61 (1.00) | 1272 | 4.49 (0.27) |
| EAS: Baseline | 52 | 64.50 (5.65) |  |  |  |  | 759 | 5.36 (1.24) | 762 | 7.11 (1.34) |  |  |
| EAS: Year 5 |  | 69.96 (5.66) | 656 | 145.70 (39.46) | 722 | 4.92 (0.34) |  |  |  |  |  |  |
| ELSA | 47 | 73.69 (9.44) |  |  |  |  | 1835 | 3.43 (1.06) | 1873 | 5.71 (1.28) |  |  |
| ET2DS: Baseline | 49 | 67.89 (4.23) |  |  |  |  |  |  | 1000 | 4.32 (0.90) | 1000 | 4.48 (0.28) |
| ET2DS: Year 1 |  | 68.91 (4.21) |  |  |  |  | 879 | 2.16 (0.68) |  |  |  |  |
| MRC NSHD | 50 | 53 |  |  |  |  | 2139 | 3.52 (0.97) | 2314 | 6.09 (1.07) |  |  |
| WHII: Phase 1 | 23 | 43.78 (5.94) |  |  |  |  |  |  | 3061 | 5.90 (1.11) |  |  |
| WHII: Phase 3 |  | 48.95 (5.98) |  |  | 2805 | 4.59 (0.35) | 3023 | 4.37 (1.00) |  |  |  |  |
| Total N |  |  | 5870 |  | 14 627 |  | 19 388 |  | 20 238 |  | 2272 |  |

Data are mean (SD). Study characteristics are given for: percentage of females in the study (F), participant’s age (Age) and number of participants available for a given trait (N). Other abbreviations are as listed above
